# Attitudes towards psychiatry amongst medical and nursing students in Singapore

**DOI:** 10.1186/s12909-019-1518-x

**Published:** 2019-03-27

**Authors:** Ellaisha Samari, Esmond Seow, Boon Yiang Chua, Hui Lin Ong, Ying Wen Lau, Rathi Mahendran, Swapna Kamal Verma, Huiting Xie, Jia Wang, Siow Ann Chong, Mythily Subramaniam

**Affiliations:** 1 Research Division, Institute of Mental Health, Buangkok Green Medical Park, 10 Buangkok View, Singapore, 539747 Singapore; 2 Psychological Medicine, Yong Loo Lin School of Medicine, National University of Singapore, Singapore, Singapore; 3 Clinical and Faculty Affairs, Duke-NUS Graduate Medical School, Singapore, Singapore; 4 Clinical Departments, East Region & Department of Psychosis, Institute of Mental Health, Singapore, Singapore; 5 Department of Nursing Administration, Institute of Mental Health, Singapore, Singapore

**Keywords:** Attitudes, Medical students, Nursing students, Psychiatry

## Abstract

**Background:**

A shortage of specialists in psychiatry, both in terms of psychiatrists and psychiatric nurses is evident worldwide. While there are multiple factors leading to an individual’s decision to specialize in psychiatry, the individual’s perceptions and attitudes towards psychiatry tend to play an essential role. This study thus aimed to explore attitudes towards psychiatry amongst medical and nursing students in Singapore and examine factors associated with these attitudes.

**Methods:**

The present cross-sectional study used an online web survey tool to assess attitudes towards psychiatry amongst 502 medical and 500 nursing students in Singapore using the Attitudes towards Psychiatry (ATP-18) scale. Descriptive statistics and multiple linear regressions were used to examine associated factors (sociodemographic and education).

**Results:**

The majority of students in this population endorsed favourable attitudes towards the following aspects of psychiatry: challenges within psychiatry, importance of psychiatry and psychiatric skills, treatment efficacy and view towards psychiatrists, but had generally unfavourable attitudes towards psychiatric patients. Male participants (compared to female; β = − 1.190, *p* < 0.05), participants in the middle income group (compared to higher income group; β = − 0.945, *p* < 0.05), participants who rated average for psychiatry lecture course and psychiatry clinical placement course (compared to above average; β = − 1.654, *p* < 0.05; β = − 1.181, *p* < 0.05) had a less favourable attitude to psychiatry. Not surprisingly, participants who were more likely to specialize in psychiatry (β = 2.053, *p* < 0.001) had a more favourable attitude towards psychiatry compared to those who were less likely to specialize in psychiatry.

**Conclusions:**

The majority of students in this study endorsed unfavourable attitudes towards patients in the psychiatric setting. The present psychiatry curriculum could be improved to nurture the development of empathetic attitudes towards people with mental illness. De-stigmatization strategies could also be integrated into other curricula besides psychiatry.

## Background

According to the World Health Organization (WHO), approximately 450 million people suffer from mental illness worldwide, with major depression being the leading cause of disability in the world [1]. Furthermore, WHO also reported that in 2014, less than 1 psychiatrist was serving 100,000 people in nearly half of the world’s population and there were approximately 7.7 nurses per 100,000 people working in mental health settings worldwide [2]. These figures have serious implications on psychiatry. A shortage of specialists both in terms of psychiatrists [3] and psychiatric nurses [4] is evident.

While there are multiple factors leading to an individual’s decision to specialize in psychiatry, the individual’s as well as public’s perception of psychiatry tend to play an essential role [5]. Prior research has brought forth several aspects of psychiatry which are negatively viewed in the literature, including its scientific background and treatment efficacy. Some studies found that as a medical discipline, psychiatry is considered not only less prestigious than other specialties [6], but also less scientific and conceptually weak [5]. Limited treatment success specifically in treating patients who do not fully recover or relapse frequently also tends to contribute to a more pessimistic view of psychiatry [7].

Furthermore, there are misperceptions and a lack of understanding about patients in the psychiatric setting which contributes to negative opinions and attitudes held towards them [8, 9]. These patients are often viewed as dangerous, unpredictable, incompetent and unlikeable [10]. Corrigan and Watson [11] found that such stigmatizing attitudes towards psychiatric patients seems to be broadly endorsed by the general public in the Western world, including Western European nations and the United States. Similar findings were also reported in studies from Asia including Singapore [12], Malaysia [13] and Japan [14]. Unfortunately, these negative attitudes extend to healthcare providers as well [15]. Notably, healthcare professionals who are taking care of psychiatric patients also experience ‘stigma by association’ in which they are subjected to similar stigmatizing stereotypes. For example, Halter et al. [16] found that psychiatric nurses were least likely to be described as skilled, dynamic and respected by their peers. Such associations have been found to correlate to more emotional exhaustion and lower job satisfaction [17] which may also contribute to psychiatry’s lack of appeal. Nevertheless, there are aspects of psychiatry that have been positively viewed upon, such as being regarded as intellectually challenging in contrast to other specialties [18].

In Singapore, medical and nursing students undergo a mandatory psychiatry rotation where they receive fundamental knowledge and clinical training in psychiatry over a course of a few weeks, with slight variations in length depending on the type of programme they are enrolled in. Regardless of their choice of eventual specialization, it is crucial that potential healthcare providers develop a favourable attitude towards psychiatry as they are likely to come into contact with patients with some form of mental illness throughout their professional life. By extension, their attitudes would affect care that people with mental health problems receive in all areas of medicine [19].

Despite its importance, there has been limited focus on attitudes towards psychiatry amongst medical and nursing students in Southeast Asia and specifically in Singapore. This study expands the literature in this area by exploring attitudes towards psychiatry amongst medical and nursing students in Singapore and examining factors associated with these attitudes.

## Methods

### Participants

The present cross-sectional study used an online web survey tool, QuestionPro® to collect data from study participants. Ethical approval was granted by the National Healthcare Group Domain Specific Review Board in Singapore. Participants for this study consisted of medical and nursing students from 2 local medical and 4 local nursing institutions in Singapore. Medical and nursing students were invited to partake in an online survey from April 2016 to September 2016 through verbal dissemination of information and sending a mass e-mail to the students by the institutional staff. Only students who were Singapore residents (Singapore Citizens or Permanent Residents) were eligible to participate. Participation was voluntary and prior to the start of the survey, participants were directed to an online consent form where they had to indicate their willingness to participate in the study. Upon completion, participants were reimbursed with a voucher to compensate them for their time. Quota was set to limit the number of participants per institution and across academic years to ensure a representative study sample.

### Design

The survey collected participants’ basic sociodemographic information, psychiatry education experience, attitudes towards psychiatry and likelihood of specializing in psychiatry.

### Attitudes towards psychiatry

The ATP-18 questionnaire is a 18-item self-report instrument that was originally developed by Wilkinson [20] to measure specific attitudes towards psychiatry, including both academic and clinical domains of psychiatry as well as attitudes towards psychiatrists and patients in the psychiatric settings. Of the 18 items, 9 (items: 2, 5, 6, 7, 11, 13, 14, 15, 17) express positive views while the other 9 (items: 1, 3, 4, 8, 9, 10, 12, 16, 18) express negative views. In this study, we adopted a 5-point Likert scale measure for each item (1 = strongly agree; 2 = agree; 3 = neutral; 4 = disagree; 5 = strongly disagree) as used in a previous study conducted by Farooq et al. [21]. Positive items were reverse coded and scores were summed, whereby the lowest possible score is 18 and the highest possible score is 90. Higher scores indicate more favourable attitudes towards psychiatry. Cronbach’s alpha in the present study was 0.621.

### Analysis

IBM SPSS Statistics Version 23 was used to perform all statistical analyses in the present study. Descriptive statistics were formulated for the overall sample, frequencies and percentages were calculated for categorical variables while means and standard deviations were calculated for continuous variables.

For descriptive analysis, responses on each item of the ATP scale were grouped according to overall responses, responses from medical students and responses from nursing students. Response items on the ATP scale were grouped according to 3 categories: ‘strongly agree’ and ‘agree’ were combined to form the ‘agree’ category, ‘neutral’ remained as a category on its own while ‘strongly disagree’ and ‘disagree’ were combined to form the ‘disagree’ category. Mann-Whitney U test was done to determine any significant differences between medical and nursing students’ mean scores for each item on the ATP scale.

Participants were also asked to report their likelihood of choosing psychiatry/psychiatric nursing on a 5-point Likert scale (“definitely decided to do”, “seriously considering”, “possible/unsure yet”, “unlikely” and “no way”) as taken from an instrument developed by Feifel et al. [22]. For descriptive analysis, responses were then grouped into binary outcomes - “yes” (comprising “definitely decided to do” and “seriously considering”) suggesting that participants were likely to choose psychiatry/psychiatric nursing as a specialty and “no” (comprising “possible/unsure yet”, “unlikely” and “no way”) suggesting otherwise.

T-tests and one-way ANOVA tests were conducted to identify differences in mean ATP scores across sociodemographic and education variables (i.e. gender, ethnicity, average monthly household income, type of student (medical or nursing), academic year(s) of study, likelihood to specialize in psychiatry, psychiatry lectures and psychiatry clinical placement experience). Multiple linear regressions were conducted to explore the associations between mean ATP score and the aforementioned variables. All statistically significant results were reported at *p* < 0.05.

## Results

### Sample

Sociodemographic characteristics of participants are summarized in Table 1. A total of 1002 medical (*n* = 502) and nursing (*n* = 500) students (28.9% males, 71.1% females) participated in this study. The mean age of participant was 21.3 years (SD = 3.3). Participants were multi-ethnic (75.2% Chinese; 14.1% Malays; 8.2% Indians; 2.5% others) and this ethnic distribution is similar to the overall ethnic distribution of Singapore Residents (74.3% Chinese; 13.4% Malays; 9.0% Indians; 3.2% others) [23]. Participants were in different years of their program (30.7% year 1; 30.9% year 2; 18.2% year 3; 20.2% year 4 and 5) and 64.6% of them reported that they had attended psychiatry lectures while 49.1% of them reported that they had attended psychiatry clinical placements. Of the 21.8% students who indicated that they were likely to specialize in psychiatry, 3% reported that they had “definitely decided to do” psychiatry and 18.8% were “seriously considering” psychiatry. Of the 78.2% students who indicated that they were unlikely to specialize in psychiatry, 35.9% reported “possible/unsure yet”, 30.7% “unlikely” and 11.6% “no way”.

**Table 1:** Sociodemographic characteristics of the study sample and likelihood of specializing in psychiatry

		*n*	%
Age	Mean = 21.27		
	SD = 3.28		
Gender	Male	290	28.9
	Female	712	71.1
Ethnicity	Chinese	754	75.2
	Malay	141	14.1
	Indian	82	8.2
	Others	25	2.5
Average monthly household income per capita over the past 1 year	Below 2000	241	24.1
	2000 to 5999	434	43.3
	6000 and above	327	32.6
Type of student	Medical	502	50.1
	Nursing	500	49.9
Academic year in course	Year 1	308	30.7
	Year 2	310	30.9
	Year 3	182	18.2
	Year 4 & 5	202	20.2
Psychiatric lecture course evaluation	Have not attended	355	35.4
	Below average	20	2.0
	Average	186	18.6
	Above average	441	44.0
Psychiatric placement course evaluation	Have not attended	510	50.9
	Below average	9	0.9
	Average	69	6.9
	Above average	414	41.3
Likelihood of specializing in psychiatry	Yes	218	21.8
	No	784	78.2

### Item responses on ATP scale

Participants’ responses on the ATP scale are provided in Table 2. Overall, participants generally endorsed favourable attitudes towards psychiatry on most of the ATP items. The majority of respondents (more than 50%) showed favourable attitudes towards 11 items (1, 3, 5, 7, 8, 10, 11, 13, 14, 15, 17), and unfavourable attitudes towards 2 items (2, 9).

**Table 2:** Item response on Attitudes to Psychiatry (ATP-18) questionnaire [20]

		Overall (%)		Medical (%)		Nursing (%)		*p* value†
		Agree	Disagree	Agree	Disagree	Agree	Disagree	
No.^a^	Items expressing positive view							
17	Empathy with patients is as important as factual knowledge in clinical practice	90.3	1.2	94.6	1.8	86.0	0.6	< 0.001
15	Psychiatric skills are essential in general practice.	87.8	0.8	93.0	0.8	82.6	0.8	0.020
7	The problems presented by psychiatric patients are often particularly interesting and challenging.	86.4	1.7	83.1	2.4	89.8	1.0	< 0.001
11	Mental illness presents us with one of the great challenges within the field of medicine.	81.4	2.0	87.8	1.2	75.0	2.8	< 0.001
13	Psychiatrists are more concerned than other doctors to establish a rapport with their patients.	75.0	7.0	76.3	8.6	73.8	5.4	0.227
5	Psychiatrists try to treat the whole patient and not just the disease.	74.9	6.9	76.7	7.2	73.0	6.6	0.394
14	Too little time is devoted to psychiatry in the medical/nursing school curriculum.	51.3	12.0	42.6	18.1	60.0	5.8	< 0.001
6	Psychiatrists are at the forefront of the movement to humanize medicine.	42.1	9.3	38.8	13.3	45.4	5.2	0.002
2	Psychiatrists are, on the whole, less dogmatic than other doctors.	14.0	60.7	9.2	69.1	18.8	52.2	< 0.001
No.^a^	Items expressing negative view							
1	Psychiatrists are often merely failed physicians.	2.0	87.2	0.8	93.8	3.2	80.6	< 0.001
10	Within medicine, psychiatry is one of the least important specialties.	10.9	71.5	5.0	83.1	16.8	59.8	< 0.001
3	Psychiatrists tend to be more emotionally unstable than other doctors.	10.9	68.3	9.2	75.3	12.6	61.2	< 0.001
8	Psychiatric patients hardly ever get better.	15.8	52.4	14.1	59.2	17.4	45.6	< 0.001
4	Psychiatrists are held in poor regard by most other doctors.	21.3	46.9	24.3	47.4	18.2	46.4	0.615
16	The practice of psychiatry is unrewarding because treatment is so lengthy and the results inconclusive.	25.3	43.0	21.9	52.8	28.8	33.2	< 0.001
12	Psychiatry is too inexact; it seems to lack a proper scientific basis.	25.0	39.5	27.7	42.6	22.4	36.4	0.764
18	Psychiatric patients, generally speaking, are not easy to like.	35.2	31.6	27.5	37.6	43.0	25.6	< 0.001
9	Psychiatric patients tend to make more emotional demands on their doctors than other patients.	62.4	11.2	71.9	10.0	52.8	12.4	< 0.001

^a^Items are arranged in decreasing order of overall percentage endorsement of positive attitudes towards the psychiatry

†Mann-Whitney U Test for comparison between medical and nursing students' mean scores for each item

### Favourable attitudes to psychiatry

The majority of participants showed favourable attitudes towards 7 out of 9 positive items. Item 17 (all students: 90.3%) had the highest endorsement of “agree” with a greater proportion of students within the medical population (94.6%) agreeing that “*Empathy with patients is as important as factual knowledge in clinical practice”* as compared to students within the nursing population (86.0%).

For the negative views, the majority of participants showed favourable attitudes towards 4 out of the 9 items. Item 1 (all students: 93.8%) had the highest endorsement of “disagree” with a greater proportion of students within the medical population (93.8%) disagreeing that “*Psychiatrists are often merely failed physicians*” as compared to students within the nursing population (80.6%).

### Unfavourable attitudes to psychiatry

The majority of participants showed unfavourable attitudes towards 1 out of the 9 positive items. Item 2 (69.1%) had the highest endorsement of “disagree” whereby a greater proportion of students within the medical population (69.1%) disagreed with the statement that “*Psychiatrists are, on the whole, less dogmatic than other doctors”* as compared to students within the nursing population (52.2%).

Out of the 9 negative items, the majority of participants showed unfavourable attitudes towards one of them. Item 9 (62.4%) had the highest endorsement of “agree” whereby a greater proportion of students within the medical population (71.9%) agreed with the statement that “*Psychiatric patients tend to make more emotional demands on their doctors than other patients*” as compared to students within the nursing population (52.8%).

### ATP-18 score

Participants had a mean score of 64.32 (SD = 5.85) on the ATP-18 scale. A score of above 60 indicates positive attitudes towards psychiatry [21]. The remaining mean scores by sociodemographic and education variables are produced in Table 3.

**Table 3:** Descriptive statistics for sociodemographic variables of ATP scores

		Mean	SD	*p* value
Overall		64.32	5.85	
Gender	Male	63.64	6.65	0.019
	Female	64.60	5.48	
Ethnicity	Chinese	64.48	5.87	0.210
	Malay	63.55	5.17	
	Indian	64.61	6.85	
	Others	63.00	5.06	
Average monthly household income per capita over the past 1 year	Below SGD2000	64.20	5.65	0.007
	SGD2000 to 5999	63.79	5.75	
	SGD 6000 and above	65.12	6.07	
Type of student	Medical	64.68	6.02	0.051
	Nursing	63.96	5.67	
Academic year in course	Year 1	64.73	5.86	0.365
	Year 2	64.00	5.64	
	Year 3	64.52	5.80	
	Year 4 and 5	64.01	6.20	
Likelihood of specializing in psychiatry	Likely	65.65	6.23	< 0.001
	Unlikely	63.95	5.69	
Psychiatric lecture course evaluation	Have not attended	65.42	5.56	< 0.001
	Below average	62.10	8.05	
	Average	62.65	5.90	
	Above average	64.25	5.77	
Psychiatric placement course evaluation	Have not attended	64.82	5.76	< 0.001
	Below average	61.22	7.00	
	Average	61.83	4.82	
	Above average	64.19	5.99	

### Correlates of ATP score

As seen in Table 3, results from t-tests and one-way Anova tests showed that five variables were significantly associated with mean ATP score on a univariate level: gender, average monthly household income, likelihood to specialize in psychiatry, psychiatry lecture and psychiatry clinical placement experience.

After controlling for other sociodemographic and education variables, only gender, average monthly household income, likelihood of specializing in psychiatry, psychiatry lecture and psychiatry clinical placement experience remained significant. This can be seen from the multiple linear regression analyses reported in Table 4 which showed the correlates of variables predicting scores of attitudes towards psychiatry. Male participants (compared to female; β = − 1.190, *p* < 0.05), participants in the middle income group (compared to the higher income group; β = − 0.945, *p* < 0.05), participants who rated average for psychiatry lecture course (compared to above average; β = − 1.654, *p* < 0.05) and psychiatry clinical placement course (compared to above average; β = − 1.181, *p* < 0.05) had a less favourable attitude towards psychiatry. Not surprisingly, participants who were more likely to specialize in psychiatry (β = 2.053, *p* < 0.001) had a more favourable attitude towards psychiatry compared to those who were less likely to specialize in psychiatry.

**Table 4:** Multiple linear regression analyses for variables predicting ATP scores

		β	95% Confidence Interval		*p* value
Age		−0.108	−0.233	0.017	0.090
Gender	Male	−1.190	−2.013	−.368	0.005
	Female	Ref			
Ethnicity	Malay	−0.942	−2.075	0.191	0.103
	Indian	−0.332	− 1.685	1.020	0.630
	Others	−1.197	−3.493	1.100	0.307
	Chinese	Ref			
Average monthly household income per capita over the past 1 year	Below SGD2000	−0.618	−1.620	0.385	0.227
	SGD2000 to SGD5999	−0.945	−1.797	−.093	0.030
	SGD6000 and above	Ref			
Type of student	Medical	0.677	−0.434	1.788	0.232
	Nursing	Ref			
Academic year in course	Year 1	0.078	−1.366	1.521	0.916
	Year 2	−0.137	−1.399	1.126	0.832
	Year 3	0.676	−0.620	1.972	0.306
	Year 4 & 5	Ref			
Likelihood to specialize in psychiatry	Likely	2.053	1.130	2.975	< 0.001
	Unlikely	Ref			
Psychiatry lecture evaluation	Have not attended	−0.405	−1.617	0.807	0.512
	Below average	−1.655	−5.753	2.443	0.428
	Average	−1.654	−3.187	−0.121	0.035
	Above average	Ref			
Psychiatry placement evaluation	Have not attended	1.072	−0.182	2.325	0.094
	Below average	−1.503	−4.275	1.270	0.288
	Average	−1.181	−2.216	−.146	0.025
	Above average	Ref			

*Ref* = reference group

## Discussion

In terms of general attitudes towards psychiatry, medical and nursing students from this study showed largely positive attitudes which is consistent with prior studies conducted in other medical and nursing populations across countries [6, 21, 24]. Specifically, the majority of students in this population endorsed favourable attitudes towards the following aspects of psychiatry (Table 2): challenges within psychiatry (item: 7, 11), the importance of psychiatry and psychiatric skills (item: 10, 14, 15, 17), beliefs about treatment outcomes (item: 8) and view towards psychiatrists (item: 1, 3, 5, 13).

### Difference between medical and nursing students’ endorsement of items on ATP-18 scale

Largely, where the majority of students endorsed unfavourably were the attitudes towards psychiatric patients. The majority of students agreed that *psychiatric patients tend to make more emotional demands on their doctors than other patients (item 9; 62.4%)* and results showed that a greater proportion of medical students (71.9%) had more unfavourable attitudes towards this item as compared to nursing students (52.8%). Negative stereotypes towards psychiatric patients are common amongst the general public which can lead to increase in fear and anxiety about working with people with mental illness [25]. Prior literature found that such stigmatizing attitudes towards people with mental illness had affected medical students’ willingness to work with them [26]. In fact, studies examining students’ experience with people with mental illness during clinical placements found that medical students had described their experience, particularly in acute inpatient setting, as stressful, intimidating and frightening [26, 27]. Furthermore, considering that this statement (item 9) emphasized the interaction between psychiatric patients and doctors, it could perhaps explain why medical students felt more strongly about this statement compared to nursing students.

Nonetheless, as prospective health care providers who are at the front line of contact with people in the community, both medical and nursing students have a pivotal role in identifying mental health issues and delivering quality care in future. Health care providers may come across people with mental health conditions in various health sectors including public health and even paediatric settings [28]. Thus, negative attitudes towards this group of patients amongst medical [26] and nursing [9] students is of concern as it may affect the way they deliver care towards these patients in future. This could lead to undesirable consequences in patient care, such as the failure of appropriate referral if patients’ needs are misunderstood, for instance in suspecting that physical complaints are imagined [29]. Prior studies have also shown that those who sought treatment have reported experiencing stigmatizing attitudes from health care providers [30] including being spoken to like a child, being given insufficient information on their conditions or treatment options, being excluded from important decisions and feeling pressurized and coerced into treatments [31, 32], causing them to feel devalued and dehumanized [33]. Thus, it is important to understand the nature of such stigmatizing attitudes and explore ways and means to improve perceptions of patients and patient care.

### Factors associated with medical and nursing students’ attitudes towards psychiatry

Findings from this study showed that students who rated their clinical placement experience and psychiatry lectures as ‘above average’ were associated with better attitudes towards psychiatry. Previously, studies have explored aspects of psychiatry education that could help to improve the image of psychiatry. Regarding attitudes towards psychiatric patients, Rusch et al. [34] found that education, and contact with psychiatric patients, are related to better attitudes towards them. In fact, prior studies showed that medical and nursing students endorsed less stigmatizing attitudes after being educated about mental illness and having contact (clinical placement) with these psychiatric patients [35, 36]. However, contrasting studies have shown that there was no change or a change in the negative direction in medical students’ attitudes towards people with mental illness following clinical placements [37]. Perhaps, these differences could be explained by variations in the psychiatric education and quality of clinical placements across programmes.

Differences in overall perceived experience amongst students in the present population could be due to various reasons. Good quality of clinical teaching, supervision, organization of clinical placement, patient contact and seeing patients respond positively to treatments during placements [38–41] have been identified as factors that create a good clinical placement experience. A study by Holm-Petersen et al. [42] also found contact experience with psychiatrists and the work psychiatrists perform to be associated with better attitudes after clinical placements, specifically in attitudes towards clinical content, effectiveness of treatment and perceived difficulty in working with patients. It was also found that encouragement received from senior psychiatrists during clinical placements is correlated to better attitudes towards psychiatry [43] and increased interest in pursuing psychiatry as a career [8]. Other factors that were found to correlate with better attitudes towards psychiatry included seeing patients respond well to treatment and receiving support from peers [43]. On the contrary, there were studies which showed that clinical placement experience may have contributed to the development of negative attitudes towards mental illness. Fisher [44] found that nursing students were at risk of developing negative attitudes in clinical placements that exposed them to situations in which they were ill-prepared for. Since differences in perceived experience of clinical placements as well as psychiatry lectures amongst students in this study sample were not examined in greater detail, we were unable to determine the factors which distinguished an ‘above average’ experience from the ‘average’ and ‘below average’ experience.

A socioeconomic status difference in attitudes towards psychiatry was found amongst medical and nursing students. Participants in the middle income group had less favourable attitudes towards psychiatry compared to the higher income group. Related research conducted on attitudes towards mental illness amongst the general population in Singapore found that a lower socioeconomic status (SES) was associated with more negative attitudes towards people with mental illness [12, 45]. Similarly, studies exploring SES difference in attitudes towards mental health care amongst students found that those with lower SES had higher perceived stigma towards it compared to those with higher SES [46, 47].

In the present study, female students from this population were significantly associated with more favourable attitudes towards psychiatry as compared to male students. However, mixed findings regarding gender difference in attitudes towards psychiatry were reported in prior studies. While some showed that females had more positive attitudes towards psychiatry as compared to males, [43, 48, 49], there were others which showed the opposite [50] citing examples that females did not consider psychiatry to be as equally respectful as other specialties and believed that psychiatrists derived less satisfaction from their work. However, considering the underrepresentation of male students in the present study sample, we were unable to draw comprehensive conclusions in this area.

As with all cross-sectional studies, the present study only provides a ‘snapshot’ of attitudes towards psychiatry amongst medical and nursing students in Singapore. Furthermore, the underrepresentation of male students is also a concern in understanding gender difference in attitudes towards psychiatry. Future research examining gender difference in attitudes towards psychiatry may consider having equitable representation from both male and female students. Notably, the relatively high percentage of neutral responses for negative items on the ATP-scale suggests the possibility of social desirability bias in their responses. Notwithstanding its limitations, this study provides important insights for medical and nursing educators who are concerned with understanding students’ attitudes towards psychiatry. It is important to note that while attitudes towards psychiatry are generally positive, the most negative attitudes are found to be towards psychiatric patients. Perhaps educators could work towards fostering greater empathy and understanding of mental health issues in their curriculum to improve attitudes towards psychiatric patients. Importantly, understanding how students learn and make sense of their clinical experience would also be helpful in applying appropriate pedagogic theory to help students overcome negative views developed during placements or after attending lectures. Educators could attempt to have an open discussion with students to identify specific components of theoretical (lectures) and clinical education (clinical placements) that are viewed more positively or negatively in order to aid the development of positive attitudes towards people with mental illness and also encourage a career in psychiatry. Additionally previous studies have commonly reported the potential of clinical placements in improving attitudes towards people with mental illness [51, 52] thereby emphasising the importance of enriching students’ experience in this regard. Hence, educators could attempt to increase students’ self-esteem and sense of competence to undergo their clinical placements before it begins by ensuring their preparedness, managing their expectations and equipping them with relevant communication skills, especially in dealing with non-adherent patients. While psychiatric placements present an opportunity to influence students positively, it is also crucial to foster these positive attitudes towards people with mental illness in their pre-clinical years and in various other curricula aside from psychiatry, especially since attitudes tend to be more amendable to change early on in training and harden as they progress through their program [53]. Essentially, the combination of accurate knowledge and contact-based strategies can help to rectify misconceptions and improve mental health literacy [54].

## Conclusion

This study found that the majority of students endorsed unfavourable attitudes towards patients in the psychiatric settings. More can be done in the present education and training curriculum to develop empathetic attitudes towards people with mental illness, as it will affect the way these prospective health care professionals deliver care towards their patients. Attitudinal interventions and de-stigmatisation strategies should be specifically focused towards selected training experiences such as in helping to prepare students for the realities of clinical placements to maximize the experience. Furthermore, de-stigmatization strategies could also be integrated into other curricula besides psychiatry, to best capture the attention of students who have negative attitudes towards people with mental illness.

## Abbreviations

ATP: attitudes towards psychiatry
WHO: world health organization
